# *MMP-8* single-nucleotide polymorphisms are related to ankylosing spondylitis in Chinese Han population

**DOI:** 10.1097/MD.0000000000012136

**Published:** 2018-08-21

**Authors:** Chenyang Meng, Rui Bai, Zhenqun Zhao, Guimei Huang, Tianbo Jin, Wei Feng, Wanlin Liu

**Affiliations:** a Department of Graduate School, Inner Mongolia Medical University, Hohhot, Inner Mongolia, China; b Department of Pediatric Orthopedics, The Second Affiliated Hospital of Inner Mongolia Medical University, Hohhot, Inner Mongolia, China; c Department of Administrative Affairs Office, The Second Affiliated Hospital of Guangxi Medical University, Nanning, Guangxi, China; d School of Life Sciences, Northwest University, Xi’an, Shaanxi, China; e Department of Pelvic and Acetabular Surgery, The Second Affiliated Hospital of Inner Mongolia Medical University, Hohhot, Inner Mongolia, China.

**Keywords:** ankylosing spondylitis, association study, genetic, *MMP-8*, single-nucleotide polymorphism

## Abstract

Ankylosing spondylitis (AS) is an extreme form of inflammatory arthritis which always leads to bony fusion of vertebral and chronic pain of back. A lot of genes including interleukin, matrix metalloproteinases (MMPs), and endoplasmic reticulum aminopeptidase were found associated with AS. MMP family members were involved in the autoimmune disease and orthopedic diseases such as rheumatoid arthritis and osteoarthritis, while few studies concentrated on the correlation between single-nucleotide polymorphisms (SNPs) in MMP and AS. In addition, there is no report on the relationship between *MMP-8* and AS. To investigate the association between SNPs in *MMP-8* and AS, we recruited 268 patients with AS and 654 healthy people to conduct a case–control study. Five SNPs including rs3740938, rs2012390, rs1940475, rs11225394, and rs11225395 of *MMP-8* gene were genotyped. It was found rs3740938 of *MMP-8* was associated with an increased risk of AS under the dominant model and additive model after adjustment for gender and age by performing logistic regression analysis (odds ratio [OR] = 1.49, 95% confidence interval [CI] = 1.02–2.18, *P* = .038; OR = 1.37, 95% CI = 1.01–1.87, *P* = .042, respectively). Moreover, haplotype “GGTCA” was associated with an increased risk of AS without adjustment for age and gender (OR = 1.75, 95% CI = 1.05–2.92, *P* = .032), while no positive result was found after adjustment for age and gender. Based on our results, our study indicates significant association between SNPs of *MMP-8* and AS risk in a Chinese Han population and these results provide the first evidence that *MMP-8* is correlated with AS.

## 1 Introduction

Ankylosing spondylitis (AS) is an extreme form of inflammatory arthritis which always leads to bony fusion of vertebral and chronic pain of back.^[1]^ The prevalence of AS in Chinese Han ethnic population was about 0.2% to 0.5%, which is similar to white Europeans and American.^[2–4]^ In addition, it often occurs in people who aged before 40, with male predominance.^[3]^ There were 3 factors involved in the pathogenesis of AS. They were environmental triggers such as microbiota and mechanical stress, autoimmune factor such as autoreactive T cells, genetic risk.^[5]^ AS is one of the most common genetic diseases and it has high monozygotic twin concordance (63%); besides, familial aggregation studies indicate a heritability of over 90%.^[6]^ It was well known that HLA-B27 played a significant role in pathogenesis of AS. However, some reports argued that it only accounted for 20% to 25% of the total heritability and 40% of the genetic risk. Fewer than 5% of HLA-B27 carriers in the general population develop disease.^[6,7]^ It suggested that there existed other genetic susceptibility. A lot of genes including interleukin (IL), matrix metalloproteinases (MMPs), and endoplasmic reticulum aminopeptidase (ERAP) were found associated with AS.^[8–10]^

The MMPs are zinc-dependent enzymes. MMPs are a family consisting of 23 protein members that could be involved in the degradation of osseous tissue and other extracellular matrices in the human body.^[11]^ It was reported that MMPs were key regulators of tissue degradation and remodeling.^[11]^ They were revealed as participating in the nosogenesis of several diseases of synovial joints such as rheumatoid arthritis (RA), osteoarthritis (OA), and AS.^[12]^ Several studies showed that AS was associated with high levels of MMP-3.^[13,14]^*MMP-8* is the member of MMPs. Just like types I, II, and III collagens, it could degrade the cartilage proteoglycan, aggrecan, and involved in tissue remodeling.^[15,16]^*MMP-8* was found to be correlated with RA and osteonecrosis of the femoral head.^[17,18]^ Mattey et al found that bath AS disease activity index (BASDAI) was correlated with a group of clustered biomarkers consisting of MMP-8, MMP-9, hepatocyte growth factor, the chemokine, and CXCL8. However, there was no study revealed the association between *MMP-8* and AS.^[19]^ In addition, the expression of *MMP-8* is related to inflammatory cytokines, growth factors, and hormones.^[20]^ As we all know, AS is inflammatory arthritis, MMP family members were involved in the autoimmune disease and some kinds of orthopedic diseases such as RA, OA, psoriaticarthritis, osteonecrosis of femoral head, and so on.^[12,21]^ The expression of MMP genes were found to be associated with several proinflammatory cytokines, such as tumor necrosis factor-alfa (TNF-α) and IL-17.^[22,23]^ Recent studies have indicated that single-nucleotide polymorphisms (SNPs) in *IL* and *ERAP* are associated with an increased risk of AS,^[24,25]^ while few studies concentrated on the correlation between SNPs in *MMP* and AS. In addition, there is no report on the relationship between *MMP-8* and AS. Based on those studies, we conducted a case–control study and genotyped 5 SNPs in *MMP* genes to investigate the association between SNPs in *MMP-8* and AS.

## 2 Materials and methods

### 2.1 Research objects

A total of 268 patients with AS and 654 healthy people were recruited among Shaanxi Province. All the subjects we recruited were the Han nationality. All patients were treated by the Xi’an Honghui Hospital and were newly diagnosed AS by clinical features and examination of laboratory and radiology. Patients who had not yet received any treatment were included for the case group. People who suffered chronic metabolic disorder of the heart, kidney, or liver and other bone diseases were excluded. Individuals with other immune or inflammatory diseases were also excluded from our study. About 654 healthy unrelated subjects were recruited randomly as control group. Individuals are Han Chinese living Xi’an. Moreover, people with chronic disease involving bone, brain, liver, heart, and lung were excluded from our study. All samples were collected with informed consent and the study was approved by the regional ethics committee.

### 2.2 SNP selection and genotyping

We reviewed the literatures related to association between *MMP-8* polymorphisms and orthopedic diseases, especially for the diseases with the similar pathologic changes as AS. In addition, SNPs associated with inflammatory response were also considered in our study. Of course, the selection of SNPs also depended on their location, allele frequencies, and disease relevance determined by use of the Hapmap public databases (dbSNP, http://www.ncbi.nlm.nih.gov/SNP/; HAPMAP, http://www.Hapmap.org/index.html.en). Finally, selected SNPs in *MMP-8* with the minor allele frequencies (MAFs) ≥5% in Asian by using HapMap database.^[17,26–28]^ In addition, the relationship between chosen SNPs and AS in Chinese Han population has not been reported before. Genomic DNA was extracted from whole blood samples using the Gold Mag-Mini Whole Blood Genomic DNA Purification Kit (version 3.0; TaKaRa, Tokyo, Japan). The DNA concentration was measured by spectrometry (DU530 UV/VIS spectrophotometer; Beckman Instruments, Fullerton, CA). The Sequenom MassARRAY Assay Design 3.0 software (Sequenom, Inc, San Diego, CA) was used to design the multiplexed SNP Mass EXTEND assay. Genotyping was performed using a Sequenom MassARRAY RS1000 (Sequenom, Inc) in accordance with the manufacturer's protocol. Sequenom Typer 4.0 software was used to perform data management and analyses.^[29]^ Based on these results, 5 SNPs including rs3740938, rs2012390, rs1940475, rs11225394, and rs11225395 were selected.

### 2.3 Statistical analysis

The differences of gender and age between 2 groups were analyzed by 2-sided Chi-squared test and independent samples t test, respectively. We performed an exact test to examine Hardy–Weinberg equilibrium (HWE) in case and control groups. Minor alleles of SNPs were seemed as risk alleles for AS susceptibility. The differences in frequency distributions of alleles were compared between cases and controls by Pearson Chi-squared test. Odds ratios (ORs), 95% confidence intervals (CIs), and *P*-value were used for logistic regression analysis and we performed the Wald test by unconditional logistic regression analysis so that the adjustment for age and sex were done for the dominant, recessive, codominant, and log-additive models. We used the Haploview software package (version 4.2) and the SHEsi software platform to analyze the linkage disequilibrium and SNP haplotypes.^[30,31]^ A logistic regression analysis was performed to assess haplotype association with response. SPSS version 22.0 statistical package (SPSS, Chicago, IL) and Microsoft Excel were used for all statistical analyses. *P* < .05 was considered statistically significant.

## 3 Result

### 3.1 Participant characteristics

In our study, we recruited 268 patients with AS and 654 healthy people. Basic characteristics of the control individuals and patients with AS are shown in Table 1. There were statistical significance differences in age between groups of case and control while no significant difference in gender.

**Table 1:**
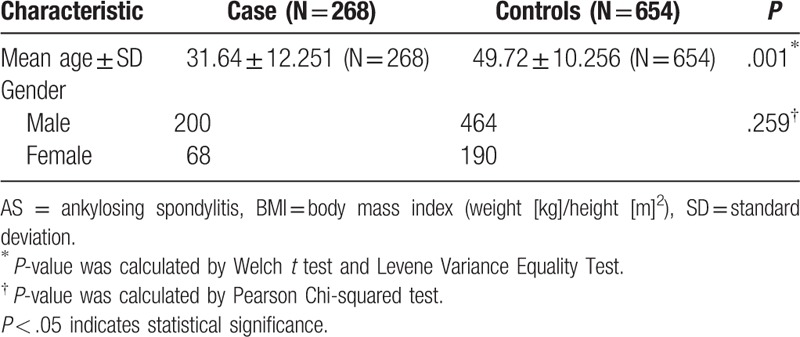
Basic characteristics of patients with AS and control individuals.

### 3.2 Hardy–Weinberg equilibrium test

Our study reveals that genotype distributions in cases and controls accorded with HWE for *MMP-8* gene rs3740938, rs2012390, rs1940475, rs11225394, and rs11225395 sites at Table 2, indicating that samples were representative.

**Table 2:**
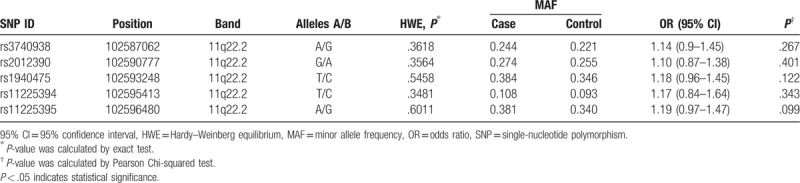
Candidate SNPs examined in *MMP-8* gene.

### 3.3 Association between genetic polymorphisms of *MMP-8* and AS risk

The detail information including position, band, MAF of candidate SNPs is summarized in Table 2. As we can see, the MAF of each SNP was >0.05. However, there was no significant difference between case group and control group in the allele model.

We further assessed the association between each chosen SNP and AS risk under 4 models including codominant, dominant, recessive, and additive model a (Tables 3 and 4). We found rs3740938 of MMP-8 was associated with an increased risk of AS under the dominant model and additive model after adjustment for gender and age by performing logistic regression analysis (OR = 1.49, 95% CI = 1.02–2.18, *P* = .038; OR = 1.37, 95% CI = 1.01–1.87, *P* = .042, respectively). Unfortunately, there was no positive result showed statistically significant difference in other SNPs we chose.

**Table 3:**
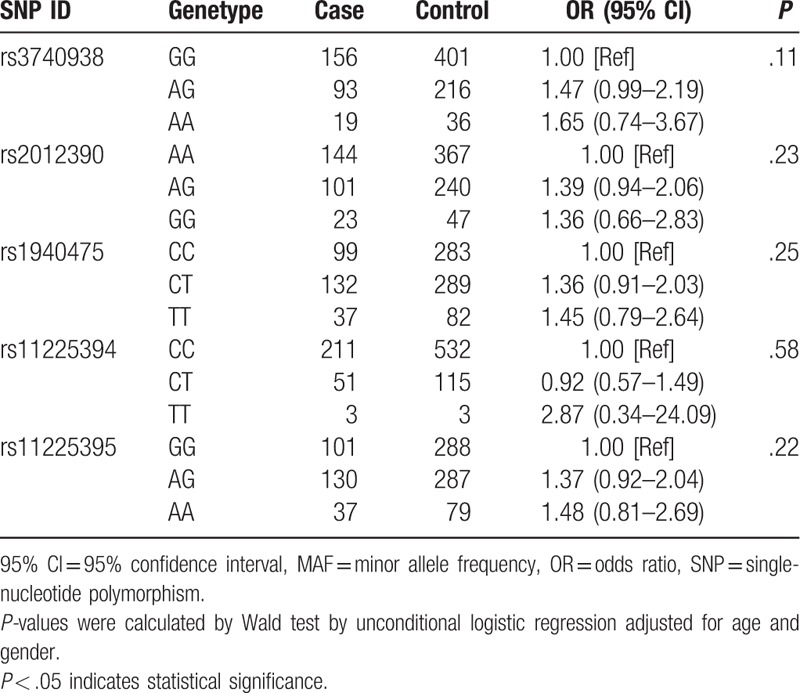
The association between the SNPs and AS risk in codominant model.

**Table 4:**
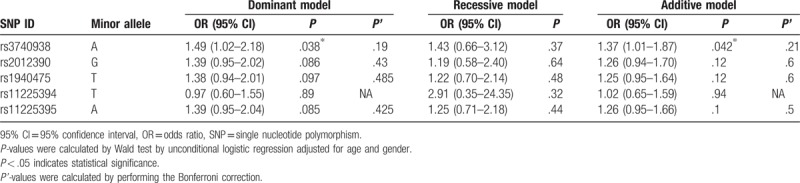
Single loci association with AS risk.

**Figure 1:**
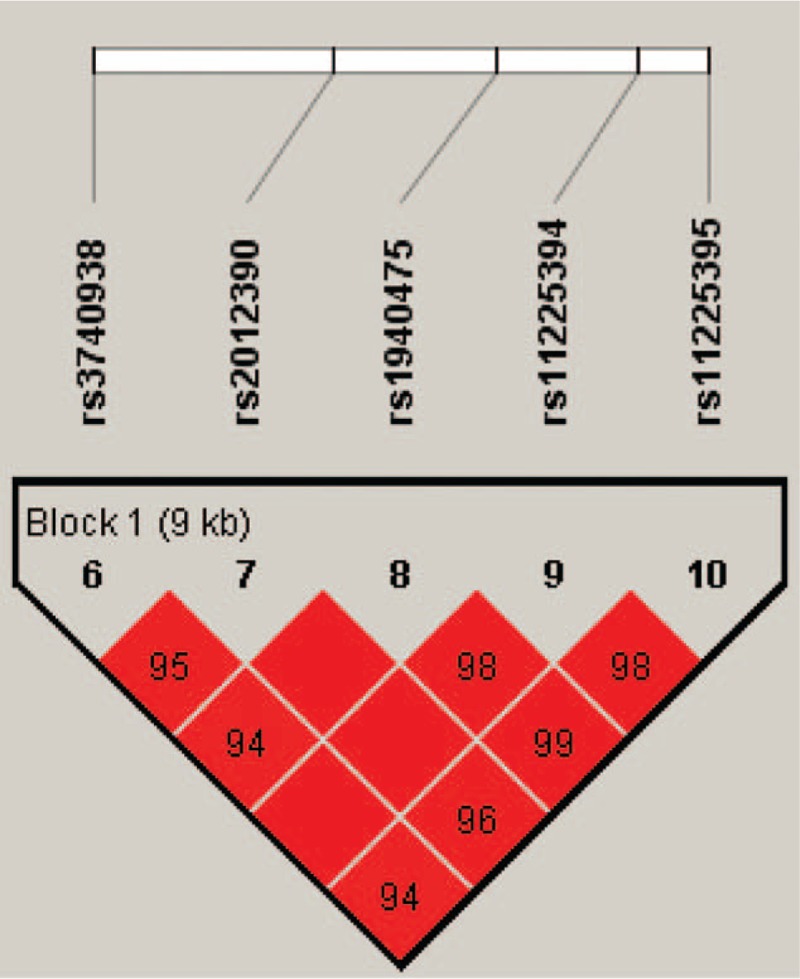
Haplotype block map and the locations for chosen single-nucleotide polymorphisms of the *MMP-8* gene.

Furthermore, there was a strong linkage between the chosen SNPs in the *MMP-8* gene (Fig. 1). We further assess the correlation between haplotype and risk of AS. As shown in Table 5, haplotype “GGTCA” was associated with an increased risk of AS without adjustment for age and gender (OR = 1.75, 95% CI = 1.05–2.92, *P* = .032). Moreover, we performed an unconditional logistic regression adjusted for age and gender while no positive result found.

**Table 5:**
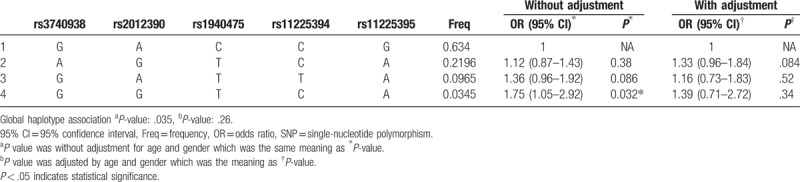
Haplotype association with response (n = 922).

## 4 Discussion

As we know, HLA-B27 is strongly correlated with AS. HLA-B27 allele was found to be a classic genetic marker for predicting the development of AS.^[32]^ Just like HLA-B27, important biomarkers such as C-reactive protein and erythrocyte sedimentation rate were associated with AS.^[32]^ Interestingly, several studies showed MMP family was evidence of biomarkers associated with AS. The study of Maksymowych et al showed high levels of serum MMP-3 could be able to predict the degree of structural damage in patients with AS.^[33]^ In addition, the gene polymorphisms on *MMP-2* and *MMP-1* were found to be correlated with AS.^[34,35]^ Although the associations between *MMP-3*, *MMP-1*, *MMP-2*, and AS were reported in many studies,^[36–38]^ there were few studies reported the correlation between *MMP-8* and AS. In our study, we found rs3740938 in *MMP-8* was associated with an increased risk of AS under the dominant model and log-additive model. We were the first study revealed the relationship between *MMP-8* and AS. Although the previous study suggested BASDAI was found correlated strongly with a component consisting of *MMP-8*, *MMP-9*, hepatocyte growth factor, and *CXCL8*, there was no research on the association between *MMP-8* and AS alone.^[19]^*MMP-8* might play a significant role in the pathogenesis of AS with or without the other MMPs involving based on our result.

The *MMP-8* gene was localized to chromosome 11q22.2. It was known as neutrophil collagenase and collagenase-2. *MMP-8* was found to be associated with RA and osteonecrosis of the femoral head.^[17,18]^ It is well known that the pathologic changes of RA and osteonecrosis of the femoral head include cartilage destruction and tissue remodeling as well as AS. In addition, MMP-8 was found to be as an important role in degrading the cartilage proteoglycan and aggrecan.^[15]^ In addition, MMP-8 was revealed as a significant role in tissue remodeling.^[16,39]^ AS is manifested as attachment point inflammation with abnormal ossification and fibrosis so that the spine become rigid. A study on the acute allergic rhinitis showed *MMP-8* may contribute to osteogenesis and fibrosis while no study revealed this result on the research of AS.^[39]^ Our study suggested gene polymorphism *MMP-8* did correlated with AS, the detail interaction between MMP-8 and AS was not clear yet.

The MMP-8 is a potent collagenolytic enzyme which is involved in the pathogenesis of several inflammatory conditions. Thirkettle and colleagues found that MMP-8 induces the expression of IL-6 and IL-8 in breast cancer cells and García et al's research showed deficiency of MMP-8 increases joint inflammation and bone erosion in the K/BxN serum-transfer arthritis model.^[18,40]^ It could be noticed that MMP-8 did affect several inflammatory factors. In fact, several cytokines such as TNF-α, interleukin-1β (IL-1β), IL-6, IL-7, and IL-8 activate signal transduction pathways to regulate *MMP* gene expression.^[41,42]^ A review reported by Malemud suggested high level of IL-6 in the sera and synovial fluid of patients with RA is likely to be responsible for the upregulation of the *MMP-9* gene as well as other MMPs that are relevant to RA as well as the degradation of cartilage proteins which is characteristic of RA pathology.^[12]^ AS is deemed as disease of immune system and the pathogenic mechanisms of AS involve several cytokines including IL-17, TNF-α, and so on.^[43,44]^ Gonzalez-Lopez et al's study reported patients with AS had higher serum TNF-α, while there was no significant difference on serum IL-6 between 2 groups.^[43]^ In addition, it was found TNF blockers inhibit spinal radiographic progression in AS by reducing disease activity.^[45]^ Our result agreed with the previous studies. Based on our result, it could be assume that cytokines regulate *MMP-8* gene expression by activating signal transduction pathways and expression of *MMP-8* reacts up on the cytokines to involve in the pathogenesis of AS. However, it should be further explored.

Just like all studies, our study has some potential limitations. First of all, this study is limited by its sample size, the further association should be confirmed finally by performing a large sample size meta-analysis. Secondly, we just collected the basic characteristics of the individuals such as age and sex. The environmental and life style factors were not included in our study but we will add those factors in further research. Furthermore, clinical characteristics including the stage of disease and detail pathologic changes were not included in our study, and it is needed to be further analyzed through additional studies. Of course, we did not perform the function study of MMP-8 due to the purpose of our study was to explore whether gene polymorphism of *MMP-8* was associated with AS. However, the detail relationship between expression of *MMP-8* gene and other cytokines will be investigated in our future study based on our present result. Last, although our result showed only rs3740938 in *MMP-8* was associated with an increased risk of AS, we revealed a relationship between *MMP-8* and AS which was not reported in previous study.

To sum up, we have demonstrated that rs3740938 in *MMP-8* gene was associated with risk of AS in Chinese Han population for the first time. Our study may provide new data for screening of AS in Han population and could be used as diagnostic and prognostic markers in clinical studies of patients with AS.

## Acknowledgments

The authors are grateful to the patients and control individuals for their participation in the study. The authors also thank the clinicians and hospital staff who contributed to sample and data collection for this study.

## Author contributions

**Conceptualization:** Chenyang Meng, Wanlin Liu, Rui Bai.

**Data curation:** Chenyang Meng, Wei Feng, Rui Bai, Zhenqun Zhao.

**Formal analysis:** Chenyang Meng, Zhenqun Zhao.

**Funding acquisition:** Rui Bai.

**Investigation:** Chenyang Meng.

**Methodology:** Chenyang Meng, Wei Feng, Zhenqun Zhao, Guimei Huang.

**Project administration:** Chenyang Meng, Wei Feng, Tianbo Jin.

**Resources:** Chenyang Meng, Rui Bai, Tianbo Jin, Guimei Huang.

**Software:** Chenyang Meng.

**Supervision:** Chenyang Meng, Tianbo Jin.

**Validation:** Chenyang Meng, Tianbo Jin.

**Visualization:** Chenyang Meng.

**Writing – original draft:** Chenyang Meng, Rui Bai.

**Writing – review & editing:** Chenyang Meng, Wanlin Liu, Wei Feng.
